# Job demands and decision control predicted return to work: the rapid-RTW cohort study

**DOI:** 10.1186/s12889-016-3942-8

**Published:** 2017-02-02

**Authors:** Lise Aasen Haveraaen, Lisebet Skeie Skarpaas, Randi Wågø Aas

**Affiliations:** 1 Stavanger Innovation Park, Olav Hansenssvei 7A, 4021 Stavanger, Norway; 2 Department of Health Studies, University of Stavanger, Postboks 8600 Forus, 4036 Stavanger, Norway; 3 Faculty of Health Sciences, Oslo and Akershus University College, Postboks 4, St. Olavs plass, 0130 Oslo, Norway

**Keywords:** Sick leave, Psychological job demands, Decision control, Sickness absence, Return to work

## Abstract

**Background:**

In order to help workers with long-term sickness absence return to work (RTW), it is important to understand factors that either impede or facilitate employee’s reintegration into the labour force. The aim of this study was therefore to examine the impact of psychological work characteristics on time-to first RTW in sick listed employees in Norway.

**Methods:**

The study was designed as a cohort study of 543 employees participating in 50 different RTW programmes. The Job Content Questionnaire (JCQ) was used to gather information on the psychological work conditions. The participants were followed for up to 18 months after they started treatment in the RTW programme. Survival analyses were used to investigate the association between psychological work conditions and time-to first RTW.

**Results:**

Having high psychological job demands (HR = .654; 95% CI: .513–.832) and low decision control (HR = 1.297; 95% CI: 1.010–1.666) were both independent predictors of delayed RTW. Employees in low-strain jobs (low demands/high control) (HR = 1.811; 95% CI: 1.287–2.549) and passive jobs (low demands/low control) (HR = 1.599; 95% CI: 1.107–2.309), returned to work earlier compared to employees in high-strain jobs (high demands/low control). No difference was found for active jobs (high demands/high control).

**Conclusion:**

This study revealed that high psychological demands, low control, and being in a high strain job reduced the probability of early RTW in sick listed employees. RTW programmes should therefore increase the focus on these issues.

## Background

Prolonged sick leave is a public health concern associated with social, health and economic consequences for the employee, as well as for the society [1]. For the individual, long-term sickness absence has been found to lead to social isolation and inactivity [2–4], depressive symptoms [3, 4], impaired self-image [2, 3], reduced well-being [5], and increased risk of disability pension [6, 7]. The annual costs related to sickness absence in Norway have been estimated to approximately 36.4 billion NOK (approx. US $6.5 billion or US $1.300 per capita) [8]. To facilitate a fast and safe return to work (RTW) is therefore of importance.

Sickness absence and work disability are complex phenomenon that can be seen as an interplay between the sick-listed employee and several factors and arenas, both at, and outside, the workplace [9, 10]. The systems and stakeholders in, or related to, the health care services and the social security systems can affect the employee and the RTW process [9, 11]. But the behaviour of the sick listed employee and how he or she copes with the disability, is also affected by physical, cognitive and emotional factors, as well as social relations at the personal level [9, 11]. In order to help employees on long-term sick leave return to work, it is crucial to understand the wide spectre of factors that either impede or facilitate employee’s reintegration to the labour force [12].

A growing amount of research has revealed that some aspects of the work environment can contribute to higher levels of sickness absence [13–16] and reduced probability of returning to work [17–21]. In addition to focusing on organizational and physical aspects of the work environment [22–25], studies also recognize the importance of psychological and social factors in a RTW process [17–21, 26, 27]. The demand-control model is one of the most widely used models for describing the impact of the psychosocial work environment on employee health. The concept of demands and control was first introduced by Karasek in 1979 [28]. Psychological job demands refer to the work pressure and workload experienced in the job, whereas decision control (or decision latitude) is concerned with the breadths of skills usable in the job and the social authority each worker has over making decisions. In the model, it is proposed that the psychological demands interact with the degree of decision control, generating four distinctly different kinds of psychosocial work experiences—also known as job types; high-strain jobs (high demands and low control), low-strain jobs (low demands and high control), active jobs (high demands and high control), and passive jobs (low demands and low control). If the demands are perceived as high and the decision control is low, job strain occurs. If, on the other hand, high demands are combined with a high level of decision control, growth, motivation and learning occurs.

An increasing amount of research has examined the association between the psychosocial work environment and work participation. However, most of the studies have used specific study groups, and different measures of RTW. This has made the results hard to generalize, and no definite conclusion has been made regarding the impact of the work environment on sickness absence and RTW. Nonetheless, the research seems to point towards an association between high job demands and delayed RTW. For example, a recent synthesis of 27 systematic reviews concluded that having high psychological job demands is a risk factor for disability and work absence [14]. Other studies suggest that low decision control and limited work flexibility affects disability and absenteeism. For example, O’Neill et al. (2010) found that low decision control reduced the RTW rates after myocardial infarction [29], and Krause et al. (2001) found that low decision control alone reduced the chances of returning to work with up to 30% for employees sick listed due to low back pain [20]. The interference of high-strain jobs on RTW has been fairly well established, and numerous studies have documented that the combination of high demands and low control reduces the probability of returning to work [17–21]. However, conflicting results have been found for the impact of the other three job types. Jansen et al. (2003) for example, found that the combination of high demands and high control (active jobs) had a positive impact on RTW [26], whereas Lidwall and Marklund (2006) found that the same combination was associated with long-term sickness absence in women [15]. These conflicting findings argue for more studies on how the psychosocial work environment affects RTW, using different populations. The aim of this study was therefore to assess the association between psychological work characteristics and time-to first RTW in a cohort of full - time sick listed employees participating in RTW programmes in Norway.

## Methods

### Design

The study was designed as a longitudinal cohort study of 543 sick listed employees participating in 50 different Rapid-RTW programmes. The study was conducted between February and December 2012. The Job Content Questionnaire (JCQ) [30] was used to gather information on the psychological work characteristics, 1 week before the programme ended. National register data on sickness absence were used to calculate time-to first RTW up to 18 months after the employees started the programme.

### Setting

The present study is one of several studies in the Rapid-RTW research project, focusing on the national rapid-RTW programme in Norway called “Raskere tilbake”[17, 31, 32]. This programme is to this date the largest effort for promoting RTW in Norway [31]. Since the programme was implemented in 2007, it has had an annual budget of NOK 700 million (approximately $ 82 million). The programme is organised by the specialist health care service and the Norwegian Labour and Welfare Administration (NAV, i.e. the directorate organising public social insurance services), and includes more than 200 different RTW services, including medical and surgical treatment in clinics, rehabilitation in hospitals (somatic), psychiatric treatment and rehabilitation, occupational training and rehabilitation in institutions, in addition to follow-up and clarification of work abilities [31]. The goal is to contribute to a faster RTW for employees on sick leave, by accomplishing more rapid clarification, medical treatment, and rehabilitation in sick leave cases. In general, there have been few guidelines for what the services should include and how they should be organised, and the content has therefore varied significantly between each service. One objection to the programme, however, has been that it does not give enough attention to workplace aspects or work characteristics.

### Study sample

A total of 920 sick listed employees were included in the study. Of these, 543 employees met the inclusion criteria of (1) being on full-time sick leave at the start of the programme; and (2) being in paid employment. For ethical reasons, we were not allowed to collect information on who declined to participate; therefore we do not have information on non-responders.

Table 1 presents the baseline characteristics of the study sample. The sample consisted of 56% women, and the mean age was 45 years (range: 21–67 SD: 9.9). Half of the participants were sick listed due to musculoskeletal disorders (53.2%). Thirty per cent of the sample had a university degree.

**Table 1:** Baseline characteristics of the study sample

Characteristic	Category	*n* ^a^	%
Gender	Male	131	22.1
	Female	332	55.9
Marital status	Married/registered partner	214	36
	Cohabiting	93	15.7
	Unmarried	82	13.8
	Divorced	58	9.8
	Separated	8	1.3
	Widowed	5	.8
Educational level	Elementary school (up to 9 years)	50	8.4
	Upper secondary school (12 years)	207	34.8
	University degree (up to 4 years)	138	23.2
	More than 4 years of university education	55	9.3
Diagnoses	Musculoskeletal	316	53.2
	Psychiatric	116	19.5
	Unspecified	41	6.9
	Cancer/tumours	41	6.9
	Nervous system	22	3.7
	Others	50	8.4
Sector	Private sector	215	36.2
	Public- Municipal level	144	24.2
	Public- Regional and governmental level	55	9.3
	Private- Publically financed sector	20	3.4
	Self-employed	7	1.2

^a^All predictors could not be assessed for every subject due to missing values

### Data collection

Each service, clinic or institution offering a Rapid-RTW programme or intervention was contacted by email and sent an invitation to participate in the study, and 50 agreed to participate. Services that agreed to participate entailed a local study coordinator, who further recruited participants to the study, 1 week before they finished treatment in the programme. Employees who agreed to participate answered self-report questionnaires concerning socio-demographic conditions, health and functioning, the services’ content, organisation and coordination, as well as various aspects of the workplace. As the interventions were independently customized for each of the employees, the length of treatment or rehabilitation varied. The stage in the RTW-process at which the employee filled in the self-report questionnaires would therefore vary.

To measure psychological job demands and decision control, a Norwegian translation of the JCQ was used [30]. Psychological job demands were measured with five items and decision control was measured with nine items. All the items were scored on a four-point Likert scale, ranging from 1 ‘strongly disagree’ to 4 ‘strongly agree’. There are several ways of calculating the sum scores of the demand-control model, however, the most common way is by using the quadrant term [33]. In accordance with this, the variables were dichotomized at the median using visual binning, in order to create high and low levels of demands and control. Values equal to the median were classified into the less hazardous exposure level, i.e. low demands or high control. The dichotomised variables were then cross-classified, creating the four job types.

The outcome measure was “days until first RTW”, measured from the day the employee started treatment in the programme until the first day the employee re-entered employment, either partially or fully. This is in line with other studies where time until first RTW is used as the outcome measure [34–36]. Employees, who had not returned to work within the follow-up time of 18 months, were censored in the analyses. Data on sickness absence was retrieved from the Norwegian Social Insurance Register. The register data were linked to the self-reported data using 11-digit personal identification numbers, retrieved from the participants in the study.

### Statistical analysis

SPSS version 21 was used for all the analysis. Survival analyses were used to calculate the time-to first RTW. Kaplan-Meier analyses were used to calculate the median time-to first RTW, whereas Cox proportional hazard analyses were used to model the effects of the independent variables on time-to first RTW. Age, gender, educational level, marital status, diagnosis, sick leave history, household income and occupational sector were entered as confounding variables in the model, as these have been shown to affect duration of sick leave in previous research [4, 37, 38]. Hazard Ratios (HRs) and 95% confidence intervals (95% CIs) were estimated for each variable in both the unadjusted and the adjusted models. Significant results were defined as *p* < .05.

## Results

Eighteen months after the employees started the programme, 77% had returned to work. The median time-to first RTW was 80 days (mean 185; SE: 196).

**Fig. 1:**
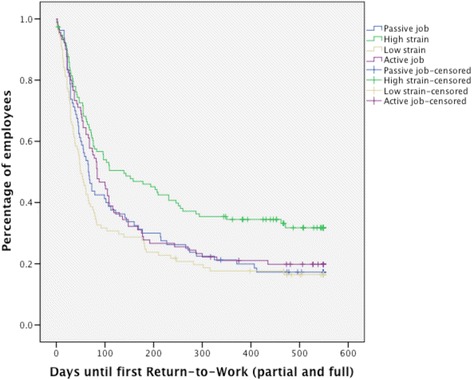
Time-to first RTW for employees in high-strain jobs, passive jobs, active jobs and passive jobs (*p* = .005)

Employees reporting high psychological job demands had lower RTW rates than employees reporting low job demands (*p* = .001), with a median of 105 days (mean: 222 days, Standard Error (SE): 15) versus 57 days (mean: 158, SE: 14), respectively. Employees who reported high decision control had significantly higher RTW rates than employees reporting low decision control (*p* = .013), with a median of 68 days (mean: 165, SE: 14) versus 102 days (mean: 218, SE: 15), respectively. Figure 1 presents the time-to first RTW for each job type. Employees in high-strain jobs (high demands and low control) had significantly lower RTW rates compared to employees in active (high demands and high control), passive (low demands and low control) and low-strain (low demands and high control) jobs (*p* = .005), with a median time-to first RTW of 207 (mean: 276, SE: 25), 79 (mean: 209; SE: 13), 65 (mean: 178; SE 25) and 50 days (mean: 170; SE: 23) for high-strain, active, passive and low-strain jobs, respectively.

Table 2 presents the results from the cox regression analyses, both unadjusted and adjusted. Having high psychological job demands (HR = .654; 95% CI: .513–.832), or being in a job with low decision control (HR = 1.297; 95% CI: 1.010–1.666) were both independent predictors of delayed RTW. Employees with low-strain jobs (HR = 1.811; 95% CI: 1.287–2.549) and passive jobs (HR = 1.599; 95% CI: 1.107–2.309) had shorter time-to first RTW compared to employees with high-strain jobs, whereas no difference was found for employees in active jobs.

**Table 2:** The association between psychological job demands, decision control, and the demand-control [39] job types on return to work, 18 months after participation in a return-to-work programme

Variable		Unadjusted				Adjusted^b^		
	*n* ^a^	HR	95% CI	*p*	*n* ^a^	HR	95% CI	*p*
Psychological job demands	405	.688	.551–.859	.001	363	.654	.513–.832	.001
Decision control	408	1.322	1.059–1.650	0.014	366	1.297	1.010–1.666	.042
The DC job types^c^	385			.005	348			004
Low-strain jobs		1.750	1.282–2.389	.000		1.811	1.287–2.549	.001
Passive jobs		1.496	.1.075–2.082	.017		1.599	1.107–2.309	.012
Active jobs		1.369	.991–1.890	.057		1.281	.887–1.849	.186

*Abbreviations*: *HR* hazard ratio, *CI* confidence interval, *DC* demand-control model

^a^all predictors could not be assessed for every subject due to missing values^. b^Adjusted for age, gender, educational level, marital status, diagnoses, previous sickness absence, workplace sector and household income ^c^ high-strain jobs were used as a reference value

## Discussion

The aim of this study was to assess the association between psychological work characteristics and time to first RTW after a RTW programme. The following results will be discussed: (1) reporting high psychological job demands increased the time-to first RTW, (2) reporting high decision control decreased the time-to first RTW, and (3) having high-strain jobs decreased the probability of returning to work early, compared to low-strain and passive jobs.

In accordance with previous studies [17, 20, 21, 27], reporting high psychological job demands was associated with delayed RTW in this study. Research on the impact of work on health has found that high job demands are not necessarily negative, in many cases high demands can lead to higher levels of motivation, learning and growth [39]. However, in a RTW situation it is possible that the work demands are perceived as extra demanding, as the employee might experience impaired job performance as a result of the disability [40, 41]. Furthermore, as high psychological demands are associated with the development of health complaints [20], it is possible that high demands induce a fear of recurring or worsening the health complaints for which one called sick to begin with, thereby reducing the chances of returning to work [26]. Consequently, having a job with high work demands might reduce the employee’s wish to return to work. A persons wish to return to work and beliefs of succeeding has been found to affect whether the employee returns to work or not [42–44]. Johansson and Lundberg (2005) suggests that whether an employee is sick listed or not is a function of the decision to go to work, and that this choice is determined not only by the persons disability, but by a function of different factors outside the individual [45]. High job demands have been linked to fear-avoidance behaviour in other studies [46], which in turn has been associated with prolonged sickness absence and delayed RTW [46–48].

Several studies have acknowledged the association between decision control and RTW [19, 20, 26, 49], and the association between low decision control and delayed RTW is well established [14, 20, 24]. These findings were further confirmed in this study. Decision control is concerned with the breadth of skills used in the job, and the social authority the employee has over using these skills to accomplish the work tasks [39]. High decision control can therefore be associated with a wider flexibility and more adjustment possibilities [4]. Having good adjustment possibilities and flexibility might make it easier for the disabled employee to regulate their work depending on their health conditions, thereby increasing the possibility of returning to work [50, 51].

Employees in high-strain jobs had decreased RTW rates compared to employees in low-strain and passive jobs. In earlier studies, low-strain jobs have been found to predict lower than average psychological strain and risk of illness [39], as the high levels of decision control allows the individual to respond to each workplace challenge optimally. In addition, as the pressure and workload is experienced as low in passive jobs, there is room for making work modifications despite the low decision control, thereby making it possible to return to work earlier than in high-strain jobs. In high-strain jobs, the employees’ decision control is low at the same time as the demands are high, restricting the adjustment possibilities in the job. Few adjustment possibilities have been found to correlate significantly with long-term sickness absence [4]. Furthermore, few adjustment possibilities restrict the use of different coping mechanisms when faced with stressful situations. As high-strain jobs are associated with excessive stress levels, remaining out of work can be considered a coping mechanism to avoid or reduce the stressful working conditions [52]. The strategy of avoidance, or restraint, is often overlooked as a potential coping strategy, as restrain from the stressor is not considered a good solution for coping with a stressful situation. However, under some circumstances it can be perceived as a necessary and functional response, as it might prevent the employee from acting prematurely and RTW before he or she is ready. As previously noted, high job demands have been linked to fear-avoidance behaviour in other studies [46], making this a plausible explanation for the delay in RTW.

Another possibility is that the work experience maintains or aggravates the employees’ ill health. High-strain jobs have been linked to impaired immune systems, and several physical disorders including musculoskeletal disorders, cardiovascular disease and even some forms of cancer [53–55]. It is thus possible that the employee’s experience of work environment directly impacts the employees’ health and recurring sickness absence [15, 27], or that high levels of work-related stress impedes for the use of adequate coping behaviours of the illness the employee experiences, extending the sickness absence period.

### Methodological discussion

This study has some limitations. The demand-control model was used as a theoretical basis for the study, and the information about work characteristics is therefore limited to the dimensions described in the model. Although the model has received a fair amount of recognition, it has also been criticised for its simplicity and lack of relevance facing the modern society’s complexity and work challenges [56]. Other work characteristics, such as attitude towards the job, job insecurity, job satisfaction and effort-reward imbalance might reveal a more complete picture of the impact of psychological work characteristics on RTW. Nonetheless, the model has achieved an increasing level of recognition in explaining an employee’s return to work in previous studies [17–21, 26, 57]. The mediating effect of social support on work-stress was not analysed in this study. According to Johnsen and Hall (1988) social support from co-workers and supervisors can function as a buffer against stress and reduce the risk of illness by increasing the employees resilience to stressors [58]. High levels of support has been found to increase the probability of returning to work early in other studies [17], and it is possible that controlling for the role of social support in a RTW process would yield different results. However, the findings in this study suggest that how the employees experience the work demands and levels of decision control does interfere with the RTW process, regardless of social support.

On should also be aware that all the predictors could not be assessed for every subject due to missing values. As the work characteristics and the confounding variables were measured through self-report, all the items in the questionnaire were not answered by all the employees. In order to correctly calculate the sum score in the JCQ, every item in the questionnaire needs to be answered, and cases with missing values can therefore not be included in the analysis. This might have impacted the results in the regression models. Of the 543 employees who were included in the study, 348 answered all the questions relevant for this study, meaning that—at most - 195 cases were lost due to missing values on either the predictor or the confounding variables.

As the work environment was measured through self-report questionnaires at the end of the treatment period, the perception of the work environment was based on recall, making it susceptible for recall-bias. However, as the study is concerned with the further development of occupational rehabilitation programmes, this is not a weakness, because the perception of the work environment in itself is likely to be relevant to the subsequent RTW process. Furthermore, as a remembered previous workplace environment can persist long after a rehabilitation programme is ended, the recalled perception of the workplace can be just as important as the actual work environment [18].

The study used a specific sample of subjects who received a RTW programme. The Rapid-RTW programme was initially intended for persons who were motivated for returning to work, and this might potentially yield different results compared to a study investigating a random sample of employees with medically certified sick leave.

## Conclusion

The results from the study indicate that having high psychological job demands and low decision control were both independent predictors of delayed RTW up to 18 months after participation in a RTW programme. Being in high-strain jobs significantly reduced the RTW rates compared to low-strain and passive jobs. This study further underpins a growing number of studies highlighting the importance of perceived work conditions in a RTW process. Identification of employees who experience their work environment as challenging is therefore important in a RTW process, as it makes it possible to meet their challenges regarding workplace issues more effectively. RTW programmes should therefore include intervention components targeting the individual and/or the work environment. We also need to test the effectiveness of such components in rigorous intervention studies.

## Abbreviations

CI: Confidence interval
HR: Hazard ratios
JCQ: Job content questionnaire
NSD: Norwegian Social Science Data Service
RTW: Return to work
SE: Standard error
